# Reduction of Connexin36 Content by ICER-1 Contributes to Insulin-Secreting Cells Apoptosis Induced by Oxidized LDL Particles

**DOI:** 10.1371/journal.pone.0055198

**Published:** 2013-01-30

**Authors:** Jacques-Antoine Haefliger, David Martin, Dimitri Favre, Yannick Petremand, Lucia Mazzolai, Amar Abderrahmani, Paolo Meda, Gérard Waeber, Florent Allagnat

**Affiliations:** 1 Service of Internal Medicine, Centre Hospitalier Universitaire Vaudois, Lausanne, Switzerland; 2 Department of Cellular Biology and Morphology, University of Lausanne, Lausanne, Switzerland; 3 Department of Physiology, University of Lausanne, Lausanne, Switzerland; 4 Service of Vascular Medicine, Centre Hospitalier Universitaire Vaudois, Lausanne, Switzerland; 5 European Genomic Institute for Diabetes, UMR 8199, University of Lille Nord de France, Lille, France; 6 Department of Cell Physiology and Metabolism, University Medical Center, Geneva, Switzerland; Indiana University School of Medicine, United States of America

## Abstract

Connexin36 (Cx36), a trans-membrane protein that forms gap junctions between insulin-secreting beta-cells in the Langerhans islets, contributes to the proper control of insulin secretion and beta-cell survival. Hypercholesterolemia and pro-atherogenic low density lipoproteins (LDL) contribute to beta-cell dysfunction and apoptosis in the context of Type 2 diabetes. We investigated the impact of LDL-cholesterol on Cx36 levels in beta-cells. As compared to WT mice, the Cx36 content was reduced in islets from hypercholesterolemic ApoE−/− mice. Prolonged exposure to human native (nLDL) or oxidized LDL (oxLDL) particles decreased the expression of Cx36 in insulin secreting cell-lines and isolated rodent islets. Cx36 down-regulation was associated with overexpression of the inducible cAMP early repressor (ICER-1) and the selective disruption of ICER-1 prevented the effects of oxLDL on Cx36 expression. Oil red O staining and Plin1 expression levels suggested that oxLDL were less stored as neutral lipid droplets than nLDL in INS-1E cells. The lipid beta-oxidation inhibitor etomoxir enhanced oxLDL-induced apoptosis whereas the ceramide synthesis inhibitor myriocin partially protected INS-1E cells, suggesting that oxLDL toxicity was due to impaired metabolism of the lipids. ICER-1 and Cx36 expressions were closely correlated with oxLDL toxicity. Cx36 knock-down in INS-1E cells or knock-out in primary islets sensitized beta-cells to oxLDL-induced apoptosis. In contrast, overexpression of Cx36 partially protected INS-1E cells against apoptosis. These data demonstrate that the reduction of Cx36 content in beta-cells by oxLDL particles is mediated by ICER-1 and contributes to oxLDL-induced beta-cell apoptosis.

## Introduction

Type 2 diabetes (T2D) originate from abnormalities in both glucose and lipid metabolism leading to β-cell failure to compensate insulino-resistance and adequately secrete the insulin necessary to maintain glucose and lipid homeostasis [1], [2].

The fine-tuning of insulin secretion in response to nutrient stimulation relies on a closely coordinated functioning of pancreatic β-cells. The importance of cell-to-cell communication mediated by gap junction channels in that process is often undervalued. In β-cells, gap junctions made solely of connexin36 (Cx36) contribute to synchronization of clusters, which appears essential to maintain β-cell function [3], [4], [5], [6] and survival [7]. We further demonstrated that long-term exposure to high concentration of glucose or saturated free fatty acids (FFAs), alone and in combination, result in a reduced expression of Cx36 in insulin-secreting cells [4], [8]. Given its key role in β-cell function and survival, Cx36 down-regulation might thus contribute to β-cell failure in relation to glucolipotoxicity.

Beside increased levels of circulating FFAs, the dyslipidemia associated with T2D is characterized by low plasma levels of High Density Lipoproteins (HDL), together with increased levels of modified atherogenic oxidized LDL-cholesterol (oxLDL) [9], [10], [11], [12], [13]. Alterations of these lipoproteins levels precede the development of diabetes and may therefore contribute to the progression of the disease [11], [14], [15]. Prolonged exposure of insulin-producing cell lines as well as isolated human and rodents islets to oxLDL particles at physiological cholesterol concentration compromises insulin production and secretion and eventually leads to β-cell apoptosis [16], [17], [18], [19]. On the other hand, HDL particles have been shown to protect the cells against harmful effects of pro-apoptotic stressors including oxLDL [20], [21]. Given its role in β-cell survival, the purpose of this study was to determine the involvement of Cx36 in the pro-apoptotic effect of oxLDL particles on β-cells. We first evaluated the impact of prolonged hypercholesterolemia on Cx36 expression *in vivo* using the hypercholesterolemic, pro-atherogenic ApoE deficient mouse (ApoE−/−) [22], [23], [24] and observed that the Cx36 levels were decreased in ApoE−/− compared to WT mice. We next studied the effect of isolated human lipoprotein particles on Cx36 expression in β-cells *in vitro* and demonstrated that prolonged exposure to oxLDL but not native LDL (nLDL) particles down-regulated Cx36 expression through a transcriptional mechanism involving the overexpression of the inducible early repressor 1 (ICER-1). We further showed that Cx36 knock-down in INS-1E cells sensitized β-cells to oxLDL-induced apoptosis and extended this observation in primary islets using Cx36 KO (−/−) mice. In contrast, Cx36 overexpression partially protected INS-1E cells from the pro-apoptotic effect of oxLDL particles.

## Materials and Methods

### Lipoprotein Preparation

Blood was collected from healthy donors. Plasma LDL fractions were isolated by sequential ultracentrifugation (LDL density, 1.063) and dialyzed against PBS. Samples were analyzed by SDS-PAGE to assess the integrity of apolipoproteins and the purity of the different fractions. The lipoprotein preparations contained less than 0.112 unit of endotoxin/µmol of cholesterol as determined by the kinetic chromogenic technique (Endotell, Allschwil, Switzerland). Oxidation of LDL particles was performed by incubation of 1 mg LDL protein/ml PBS with 5 µM CuSO4 at 37°C for 6–8 h. The oxidation reaction was stopped at 4°C for 30 min by adding 300 µM EDTA. The oxidized as well as native LDL particles were dialysed against PBS and subsequently against either DMEM or RPMI medium without foetal calf serum. The oxidation reaction was verified by determining the lipid peroxide content as previously described [19].

### Cell Culture

The rat insulinoma cell line INS-1E (kindly provided by Dr. Pierre Maechler, CMU, University of Geneva [25]) was maintained in the complete RPMI 1640 medium as previously described [25]. MIN6-B1 (kindly provided by Dr. Philippe. Halban, CMU, University of Geneva [26]) were cultured in DMEM as previously described [26].

### Oil Red O Staining

Oil red O (Solvent Red 27 or Sudan Red 5B) was used to stain endogenous lipid deposits. INS-1E cells grown or glass coverslips were fixed for 30 min in 3.7% paraformaldehyde. After washing, fixed cells were incubated for 20 min in oil red O (Sigma-Aldrich) staining solution (0.5% in isopropanol), and counter-stained lightly by dipping the coverslips in an hematoxyline solution [27].

### Mouse Models and Langerhans Islets Preparation

Rat or mouse islets of Langerhans were isolated from the pancreas by collagenase digestion, filtered on a 100 µM cell strainer (BD Biosciences), hand-picked under a stereomicroscope and cultured as previously described [4], [7], [28]. WT or ApoE−/−4 months old male C57BL6 mice were generated, housed and cared for as previously described [23], [29]. Blood samples for plasma analyses were taken by heart puncture. Concentrations of glucose and lipids were measured in the plasma of mice sacrified while in a fed state. Measurements were conducted at The Mouse Metabolic Evaluation Platform facility from the Universty of Lausanne (http://www.cardiomet.ch/en/cmet_home/cardiomet-chercheurs/cardiomet-chercheurs-plateforme_metabolique.htm). The Cx36−/− mice were generated, housed and cared for as previously described [7], [28].

### Western Blot Analyses

Cells were washed once with cold PBS and directly lysed with Laemmli buffer. Nuclear extracts for ICER-1, ICER-1γ and CREM immunoblots were prepared as previously described [4]. Lysates were then resolved by SDS-PAGE and transferred to a PVDF membrane. Immunoblot analyses were performed as previously described [4], [30] using the following antibodies: Rabbit polyclonal antibodies against Cx36 [4], [8], monoclonal anti α-tubulin antibodies (Fluka Chemie, diluted 1∶2,000); rabbit polyclonal anti CREM-1 sc440 (Santa Cruz, 1∶500). After incubation at room temperature (1 h) with the appropriate secondary antibody conjugated to horseradish peroxidase (Fluka Chemie, diluted 1∶20,000), membranes were revealed by enhanced chemiluminescence (immobilon, millipore) using the ChemiDoc™ XRS+ System and analyzed using the accompanying proprietary program Image Lab (BETA2) version 3.0.01(Bio-Rad Laboratories, Reinach, Switzerland ).

### RNA Isolation, and Quantitative RT-PCR (Lightcycler©)

Cells were homogenized in the Tripure Isolation Reagent (Roche Diagnostics) and total RNA was extracted using the kit procedure. mRNA from freshly isolated mouse islets were isolated using nucleospin RNA II columns (Macherey-Nagel). Transcripts (1 µg) were reverse-transcribed using ImProm-2 Reverse transcription System (Promega). Quantitative PCR was performed using the SYBR® Premix ExTaq™ (Takara) in a Lightcycler Instrument (Roche Diagnostics). cDNAs were amplified using the following primers: rat Cx36: 5′-ATACAGGTGTGAATGAGGGAGGATG-3′ (sense); 5′- TGGAGGGTGTTACAGATGAAAGAGG-3′ (antisense). Rat ribosomal protein L-27 5′-GATCCAAGATCAAGTCCTTTGTG-3′ (sense); 5′-CTGGGTCTCTGAACACATCCT-3′(antisense). Rat ICER-1: 5′-CTGGGTCTCTGAACACATCCT-3′ (sense) 5′-CACCTTGTGGCAAAGCAGTA-3′(antisense). Rat Plin ′-GCTTCTCTCCCCAAGGAAAC-3′ (sense); 5′-TGCCCCTTAAAACCTGACTG-3′ (antisense). Rat ACC1 5′-CAGGTTCAGAGCGAGAGATG-3′ (sense); 5′-ATGATGGCTCGGATGAAGAA-3′ (antisense). Rat SOD1:5′-TTCCATCATTGGCCGTA-3′ (sense); Rat SOD1: 5′-AAGCGGCTTCCAGCATTTC-3′ (antisense). Rat-SOD2: 5′-TGGTGTGAGCTGCTCTTGATTG-3′ (sense); Rat SOD2: 5′-GCCCCAACACAGAGATGGAATA-3′ (antisense).

### Transient Transfection and Luciferase Assays

INS-1E cells were co-transfected using lipofectamine 2000 (Invitrogen, Baesley, UK) with the internal control pRL-CMV encoding Renilla luciferase (Promega, Madison, WI, USA), various reporter plasmids containing the luciferase gene under control of different fragments of the human Cx36 promoter, together with an empty vector (pCDNA3), or a plasmid allowing constitutive expression of ICER-1 [31] or an ICER antisense plasmid [32], as previously described [4], [8]. 24 hours after transfection, the cells were incubated in presence or absence of 2 mM (78 mg/dl) nLDL or oxLDL particles. Sample preparation, luciferase activities and values correction were performed as previously described [4], [8].

Rat Cx36 *Silencer®* Select pre-designed siRNA s132237 (siRNA#1) and s132238 (siRNA#2) were from Applied Biosystems (Life Technologies Corporation, Carlsbad, California, U.S.A). The Allstars Negative Control siRNA (Qiagen, Hombrechtikon, Switzerland), which has no effect on β-cell gene expression and viability, was used as a control [30], [33], [34]. siRNA transfections were conducted as previously described using lipofectamin RNAiMax (Invitrogen) with a final concentration of 30 nM siRNA. The efficiency of transfection is >90% [30], [33], [34]. siRNA transfections under these conditions do not affect β-cell function [30], [34]. Cells were then cultured for a 48-hour recovery period before being collected or treated as indicated.

### ROS/RNS Superoxide Measurements

The ROS/RNS and superoxide production were measured using the total ROS/Superoxide Detection Kit (Enzo Life Sciences AG, Lausen, Switzerland). Briefly, INS-1E cells were plated in 96 well plates. Following a 48 h treatment with lipoproteins, cells were washed once and incubated for 30 min in 100 µl of wash buffer containing 2 µM of oxidative stress detection reagent (green) and 2 µM of superoxide detection reagent (orange). Fluorescence was quantified using a fluorescence microplate reader and standard fluorescein (Ex = 488 nm, Em = 520 nm) and rhodamine (Ex = 550 nm, Em = 610 nm) filter sets.

### Generation of Recombinant Adenoviruses and Cell Infection

Control adenovirus encoding GFP (Ad-GFP) was generated as previously described [35]. The adenovirus encoding rat Cx36 (Ad-Cx36) was generated by Vector Biolabs, Philadelphia, PA, U.S.A. using rat the complete rat Cx36 coding sequence (GenBank: AJ296282.1) cloned using the TA cloning system pGEM-Teasy (Promega). Infection was performed as previously described [5], [30].

### Assessment of Cell Viability

The percentage of viable, apoptotic and necrotic cells was determined using the DNA-binding dyes Propidium Iodide (PI, 5 µg/ml) and Hoechst 33342 (HO, 5 µg/ml, Sigma-Aldrich) [36]. The cells were examined by inverted fluorescence microscopy (Axiovert 200, Carl Zeiss, Zaventem, Belgium). A minimum of 500 cells was counted in each experimental condition by two independent observers, one of them unaware of sample identity. Total islet viability was evaluated by two independent observers, both of them unaware of sample identity. At least 20 islets per condition were counted, as previously described [37].

### Statistical Analysis

Data are presented as means ± SEM. Comparisons were performed by two-tailed paired Student’s *t*-test or by one-way ANOVA followed by t-tests with Bonferroni correction for multiple comparisons. Non-parametric χ2 tests were further used to assess differences between non-Gaussian distributions. A *p* value ≤0.05 was considered statistically significant.

### Ethics Statement

Mouse care, surgery and euthanasia procedures were approved by our institution and the Cantonal Veterinary Office (Service de la Consommation et des Affaires Vétérinaires SCAV-EXPANIM). Written, informed consent was obtained from all blood donors. The study protocols for blood collection and lipoproteins preparation were reviewed and approved by the clinical research ethics committee of the Centre Hospitalier Universitaire Vaudois (CHUV).

## Results

**Figure 1:**
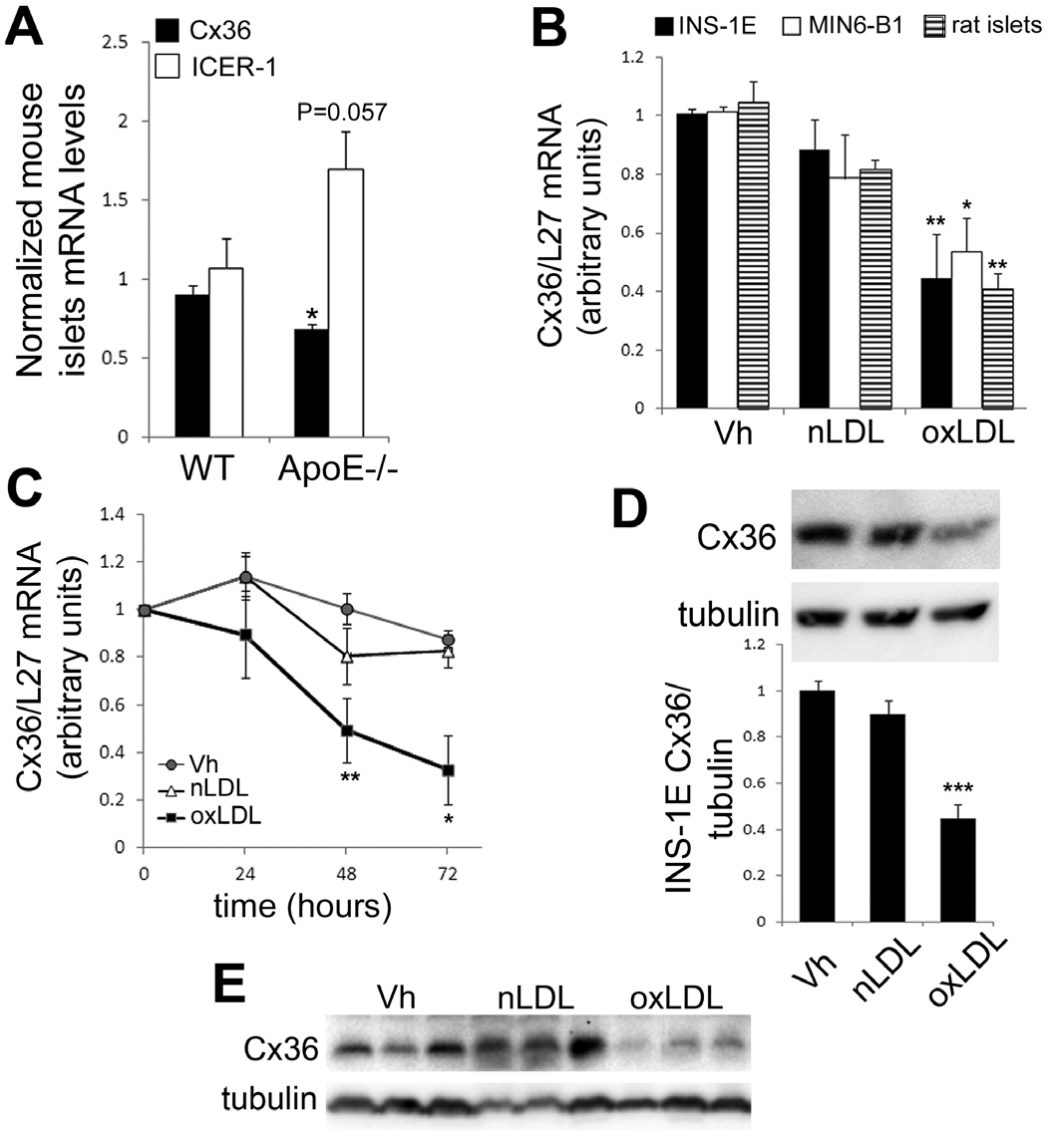
Prolonged exposure to oxidized LDL particles decreases Cx36 levels in β-cells. A) Langerhans islets were isolated from adult male WT or ApoE−/− C57Bl6 mice and immediatly sampled for mRNA extractions. Cx36 and ICER-1γ mRNA levels were analyzed by quantitative RT-PCR, and normalized to the expression of the housekeeping gene L27. Results are means ± SEM of six animals in each group. B) INS-1E cells, MIN6-B1 cells, or primary rat islets were cultured for 72 h in presence or absence (Vh for Vehicule) of 2 mM native (nLDL) or oxidized LDL (oxLDL). Quantitative RT-PCR of Cx36 mRNA levels normalized to the levels of the housekeeping gene L27. Data represent mean ± SEM of four to six independent experiments. *P<0.05; **P<0.01 vs. control. C) Time course analysis of Cx36 mRNA levels in INS-1E cells cultured in absence (Vh for Vehicule) or presence of 2 mM native (nLDL) or oxidized LDL (oxLDL). Data are mean ± SEM of four independent experiments. *P<0.05; **P<0.01 vs control condition (vehicle treated). D–E)Western blot analyses of Cx36 levels in INS-1E cells (D) or primary isolated rat islets (E) cultured for 48 h in presence or absence (Vh for Vehicule) of 2 mM native (nLDL) or oxidized LDL (oxLDL). (D) Data are mean ± SEM of five independent experiments. (E) Blot shows three independent experiments.

To assess whether cholesterol participates *in vivo* to the control of Cx36 expression, we characterized the expression of Cx36 in ApoE−/− C57BL6 male mice. As compared to WT mice, ApoE−/− mice displayed an 8 fold increase in total circulating levels of cholesterol and a 70 fold increase in LDL levels (Table 1). In addition there was also a 3 fold increase in triglycerides and a 2 fold increase in circulating FFA, whereas the glycemia remained normal and similar in both groups (Table 1). As compared to WT mice, Cx36 mRNA levels were significantly decreased in ApoE−/− mice, and there was a trend toward an increase in ICER-1 mRNA level (P = 0,057; Figure 1A). Moreover, quantitative assessment of Cx36 immunofluorescent labeling on frozen pancreas sections (see Methods S1) from WT or ApoE−/− mice revealed a significantly decreased Cx36 punctate staining in ApoE−/− compared to WT mice (Figure S1).

**Table 1:** Characteristic of WT and ApoE−/− mice.

	WT	ApoE−/−
**Weight (gr)**	25.82±0.53	28.9±0.52*
**Glucose (mg/dl)**	182.83±14.97	206.23±16.34
**TG (mg/dl)**	90.64±8.96	316.53±28.68***
**FFAs (mg/dl)**	13.79±2.89	27.18±3.77*
**Total cholesterol (mg/dl)**	75.02±3.03	316.53±38.83***
**VLDL/LDL (mg/dl)**	4.90±0.62	370.51±26.27***
**HDL(mg/dl)**	58.52±2.41	109.0±3.99***

Weight, plasma glucose, triglycrides (TG), free fatty acids (FFAs), total cholesterol, low density lipoprotein (LDL) and high density lipoproteins (HDL) levels of adult male WT or ApoE−/− C57Bl6 mice. Data are means ± SEM of 8 animals in each group. *P<0.05; ***P<0.001 vs. WT group.

We then investigated the *in vitro* effects of human native (nLDL) or oxidized LDL (oxLDL) on Cx36 expression in insulin-secreting cell lines and primary rat islets. Cx36 mRNA levels were decreased by 50% in INS1-E cells, MIN-6B1 cells and primary rat islets cultivated for 72 hours in medium supplemented with 2 mM (78 mg/dl) of human oxLDL particles, but not nLDL (Figure 1B). Time course analysis revealed that Cx36 mRNA expression was already significantly reduced after 48 h of culture in presence of oxLDL (Figure 1C). Subsequent experiments were performed after 48 h of exposure to lipoproteins. At the protein level, Cx36 expression was decreased in both INS-1E cells (Figure 1D) and primary isolated islets (Figure 1E) exposed for 48 h to 2 mM oxLDL, but not nLDL.

**Figure 2:**
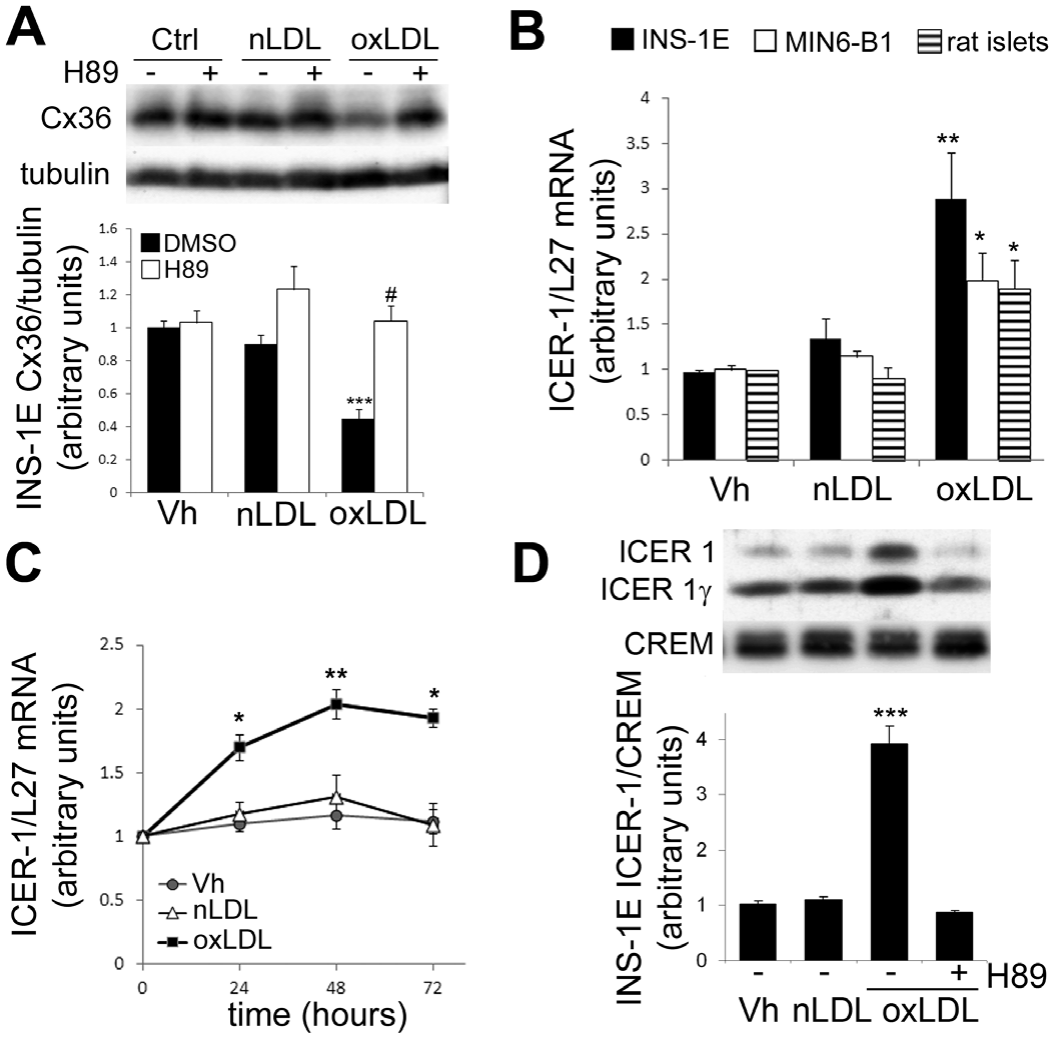
oxLDL overexpress ICER-1 and downregulate Cx36 in a PKA-dependent manner. A)Western Blot analyses of Cx36 over tubulin levels in INS-1E cells cultured for 48 h in presence or absence (Vh for Vehicule) of 2 mM native (nLDL) or oxidized LDL (oxLDL), together or not with the PKA inhibitor H89 (10 µM). *Upper panel:* representative Western blot; *lower panel*: means ± SEM of three independent Western blots. ***P<0.01 vs. INS-1E control. #P<0.05 vs. oxLDL condition in absence of H89. B) Quantitative RT-PCR of ICER-1 over L27 mRNA expression in INS-1E cells, MIN6-B1 cells, or primary rat islets cultured for 72 h in presence or absence (Vh for Vehicule) of 2 mM native (nLDL) or oxidized LDL (oxLDL). Data represent mean ± SEM of four to six independent experiments. C) Time course analysis of ICER-1 mRNA levels in INS-1E cells cultured in presence of oxLDL. *P<0.05; **P<0.01 vs control condition (vehicle treated). D) Western Blot analyses of ICER-1 and ICER-1γ levels in INS-1E cells cultured for 48 h in presence or absence (Vh for Vehicule) of 2 mM native (nLDL) or oxidized LDL (oxLDL), together or not with the PKA inhibitor H89. *Upper panel:* representative Western blot; *lower panel*: Results are means ± SEM of three independent Western blots. ***P<0.01 vs. INS-1E control.

We previously showed that Cx36 expression is controlled by the cAMP/PKA pathway [4], [8]. INS-1E cells were exposed to nLDL or oxLDL for 48 h, in presence of the cAMP dependent protein kinase A (PKA) inhibitor H89 (10 µM). As shown in Figure 2A, H89 prevented the Cx36 decrease elicited by oxLDL, suggesting that the cAMP/PKA pathway mediates the oxLDL effect on Cx36 expression. We recently established that ICER-1 is overexpressed after prolonged exposure to oxidized, but not native, LDL particles [19]. Here, we confirmed that oxLDL, but not nLDL particles, induced a two to three fold increase in ICER-1 mRNA levels in INS-1E cells, MIN-6 cells and isolated rat islets (Figure 2B). Time course analysis further revealed that ICER-1 mRNA expression is already significantly upregulated after 24 h of culture in presence of oxLDL (Figure 2C). Using an antibody directed against CREM-1 (cAMP response element modulator) that detected ICER-1 and ICER-1γ, the two major repressive isoforms of CREM expressed in β-cells, we further confirmed that a 48 h exposure to oxLDL particle, but not nLDL particles, increased ICER-1 and ICER-1γ protein levels in nuclear extracts from INS-1E cells (Figure 2D). In addition, the oxLDL-driven ICER-1 overexpression was PKA-dependent as H89 inhibited ICER-1 overexpression in nuclear extracts from INS-1E cells (Figure 2D).

**Figure 3:**
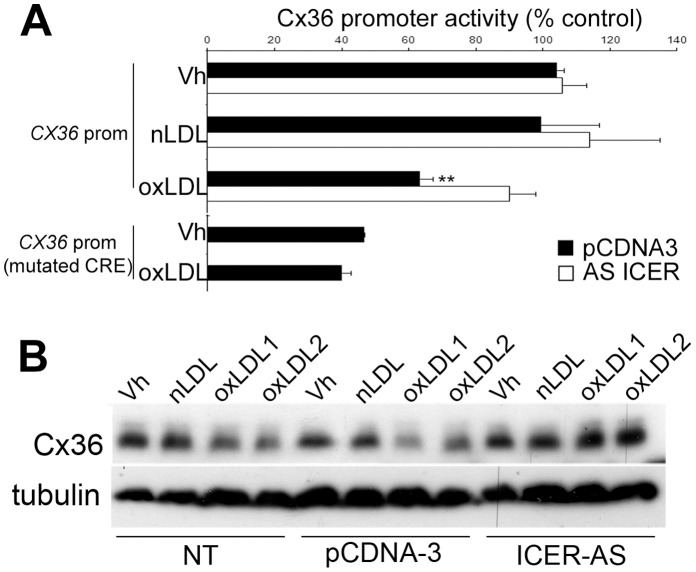
ICER-1 overexpression mediates the effect of oxLDL on Cx36 expression. A) INS-1E cells were cotransfected with a plasmid expressing the reporter gene luciferase under the control of a 1 kb fragment of the *CX36* promoter, or a fragment containing a mutated CRE, together with an empty vector (pCDNA3; black bars) or an antisense ICER plasmid (ICER AS; white bars). 24 h post transfection, cells were cultured for 48 h in presence or absence (Vh for Vehicule) of 2 mM nLDL or oxidized LDL oxLDL. Cx36 promoter activity was evaluated by luciferase assay. Data are mean ± SEM of five to six experiments. **P<0.01 vs. vehicle-treated cells. B) Western Blot analyses of Cx36 over tubulin levels in INS-1E cells non transfected (NT), transfected with an empty vector (pCDNA3) or the antisense ICER plasmid (ICER AS) and treated with native LDL (nLDL) or two different preparations of oxidized LDL (oxLDL1 or 2). Data are representative of three independent experiments.

We have previously demonstrated that ICER-1 binds to a highly conserved CRE (cAMP response element) located between bases −566 and −556 upstream of the transcription start site of the Cx36 gene [4], [8]. To assess the involvement of this CRE in the oxLDL-induced Cx36 downregulation, a plasmid expressing the reporter gene luciferase under the control of a fragment of the human CX36 promoter containing the CRE (pGL3-1079) was transiently transfected in INS-1E cells incubated in presence of 2 mM LDL, native or oxidized, for 24 hours. oxLDL induced a 40% decrease in the luciferase activity driven by the CX36 promoter fragment (Figure 3A). In contrast, oxLDL did not reduce the activity of a similar plasmid containing a mutated CX36 CRE (pGL3-1079m). To investigate the involvement of ICER-1 in the control of the CX36 gene expression, INS-1E cells were cotransfected with either an empty vector (pCDNA3) or an antisense ICER construct (ICER AS) reducing the endogenous ICER-1 and ICER-1γ content [4], [32]. ICER AS fully blocked the effect of oxLDL on the CX36 promoter activity, indicating that ICER-1 drove the oxLDL effect on Cx36 expression. Furthermore, blocking ICER-1 overexpression using ICER AS prevented oxLDL-induced down-regulation of endogenous Cx36 protein (Figure 3B).

**Figure 4:**
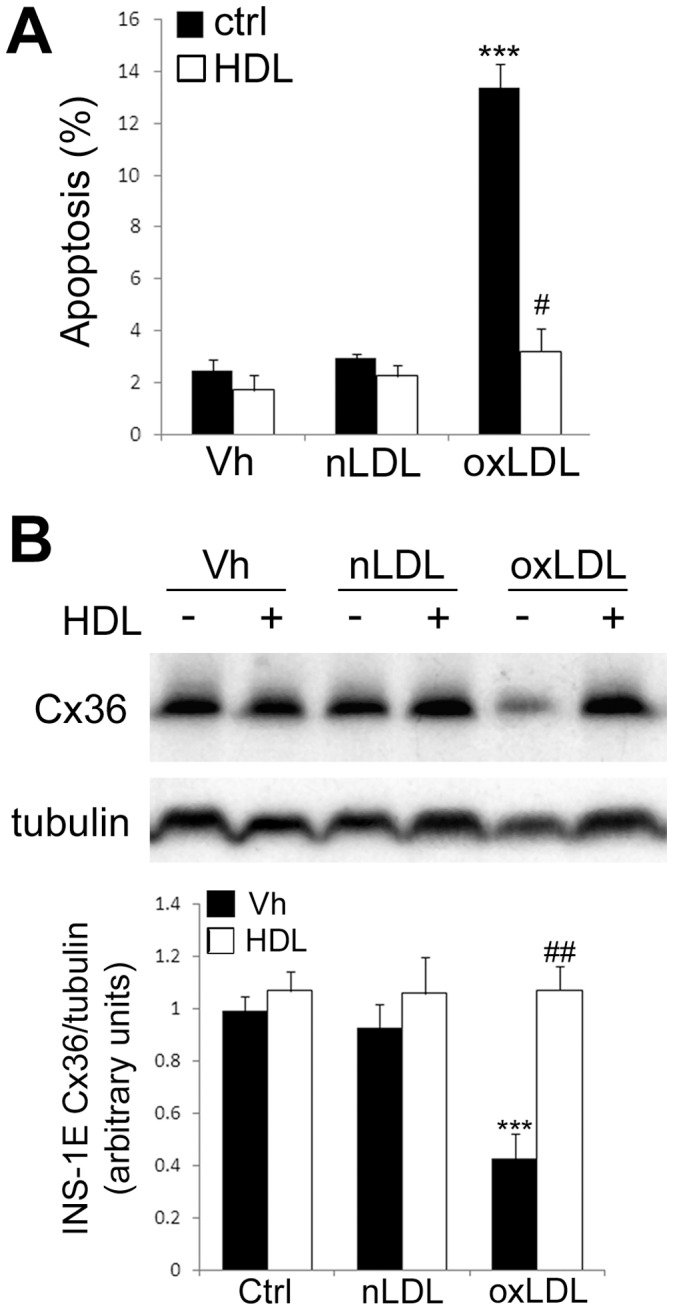
HDL prevent the deleterious effect of oxLDL particles on INS-1E cells survival and Cx36 expression. INS-1E cells were cultured for 48 h in presence or absence (Vh for Vehicule) of 2 mM nLDL or (oxLDL, in presence or not of 0.5 mM HDL. (A) Apoptosis levels were evaluated by HO-PI staining. (B) *upper panel.* Representative Western Blot anti-Cx36 and tubulin. *Lower panel:* quantitative assessment of four independent WB. A–B) Data are means ± SEM of four independent experiments. ***P<0.001 vs. control. ^#^P<0.05; ^##^P<0.01 HDL-treated cells vs. oxLDL-treated cells.

We recently demonstrated that Cx36 plays a protective role in cytokine-induced β-cell apoptosis [7] and prolonged exposure to oxLDL induces β-cell apoptosis [17], [19]. Here, we observed that a 48 h exposure to 2 mM oxLDL induced 15 to 20% apoptosis in INS-1E cells and we confirmed that 1 mM (40 mg/dl) HDL particles protected β-cells from oxLDL-mediated apoptosis [17], [18] (Figure 4A). HDL particles also prevented the oxLDL-induced Cx36 protein downregulation (Figure 4B), indicating that there is a correlation between the decreased levels of Cx36 and oxLDL-induced apoptosis.

**Figure 5:**
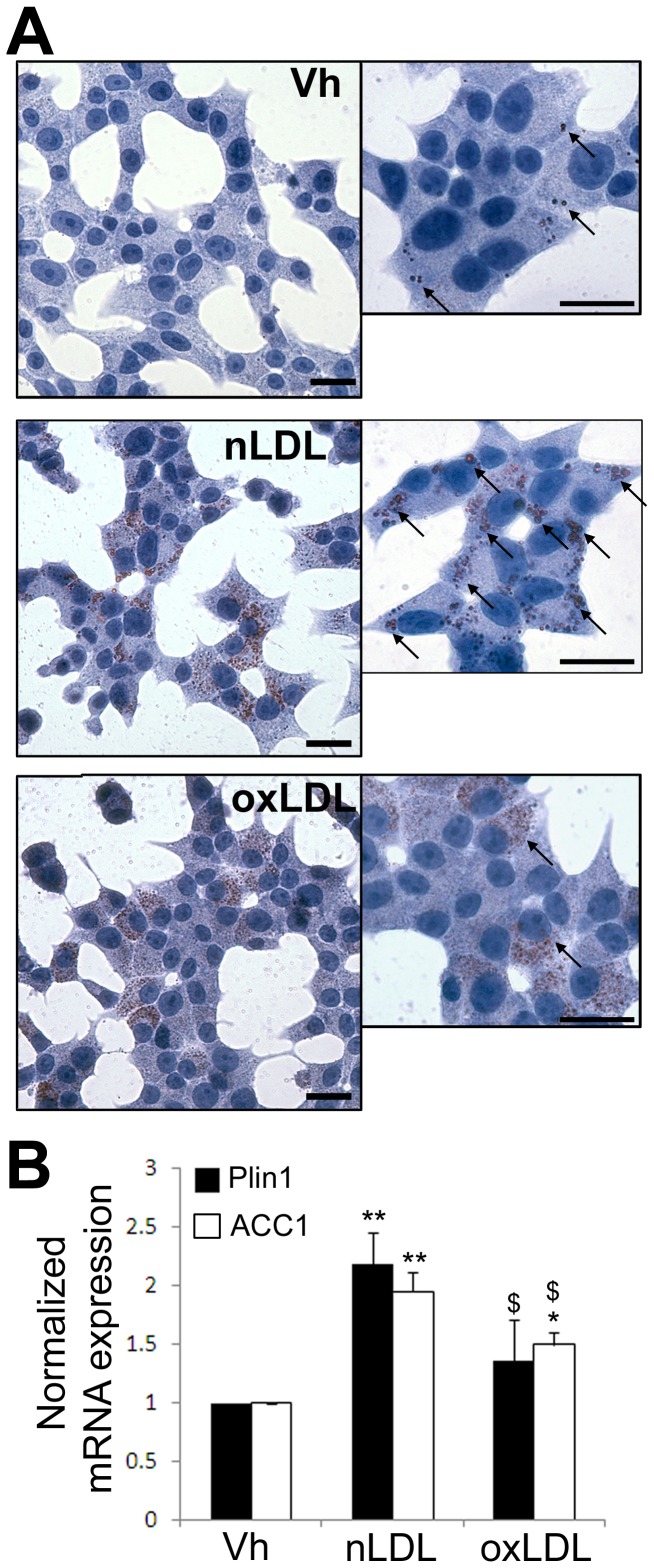
nLDL but not oxLDL particles are converted in lipid droplets in INS-1E cells. (A) Oil Red O staining of INS-1E cells cultured for 24 h in presence or absence (Vh for Vehicule) of 2 mM nLDL or oxLDL particles (black bar = 10 µm). Arrows point to lipid droplets. (B) Quantitative assessment of Plin1 (black bars) and ACC1 (white bars) over L27 mRNA expression in INS-1E cells cultured for 48 h in presence of 2 mM nLDL or oxLDL particles. *P<0.05, **P<0.01 vs. Vh condition. ^$^P<0.05, vs. nLDL condition.

**Figure 6:**
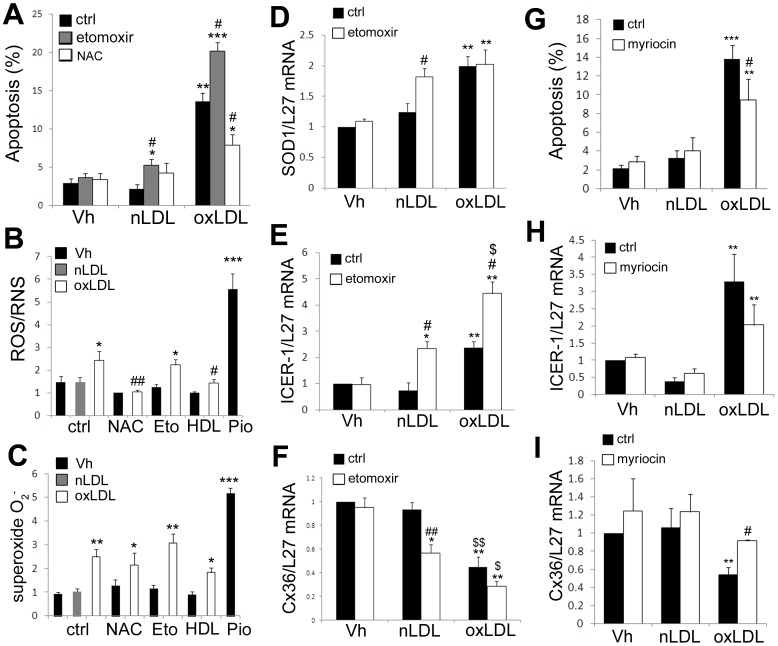
nLDL but not oxLDL particles are β-oxidized in INS-1E cells. (A, G) INS-1E cells were cultured for 48 h in presence of 2 mM nLDL or oxLDL particles, together or not with etomoxir (50 µM), NAC (1 mM) or myriocin (100 nM). Apoptosis levels were evaluated by HO-PI staining. (B–C) INS-1E cells were cultured for 48 h in presence of 2 mM nLDL or oxLDL particles, together or not with NAC (1 mM), HDL (1 mM), etomoxir (Eto:50 µM). As a positive control, cells were treated for 30 min with piocyanin (Pio:200 µM). Data are means ± SEM of five live cells measurements of ROS/RNS (B) and superoxide O_2_^−^ (C) production. (D–I) INS-1E cells were cultured for 48 h in presence of 2 mM nLDL or oxLDL particles, together or not with etomoxir (50 µM) or myriocin (100 nM). RT-PCR analysis of SOD1 (D), ICER-1 (E, H) and Cx36 (F, I). *P<0.05, **P<0.01, ***P<0.001 vs. Vh condition. ^$^P<0.05, ^$$^P<0.01 vs. nLDL condition. ^#^P<0.05; ^##^P<0.01 vs. respective condition without etomoxir, NAC, HDL or myriocin.

Lipid toxicity in β-cells is thought to be mostly due to accumulation of free fatty acyl CoA levels entering non-oxidative toxic pathways of fatty acids metabolism, such as de novo ceramide formation which trigger reactive oxygen species (ROS) production [38]. This may be caused both by a defect in lipid storage in the form of neutral lipid droplets, or through a defect in lipid β-oxidation in the mitochondria. We monitored the formation of lipid droplets after a 24 h exposure to 2 mM nLDL or oxLDL particles in INS-1E cells. In control condition, rare small regular lipid droplets were detected whereas abundant big regular lipid droplets were observed in presence of nLDL. In contrast, in presence of oxLDL, the lipid droplets were smaller and less abundant, suggesting that oxLDL were not stored in lipid droplets to the same extend as nLDL (Figure 5A). Exposure to nLDL also stimulated the expression of the perilipin 1 (Plin1) transcripts, a marker of lipid droplets, whereas oxLDL treatment did not significantly increase Plin1 mRNA expression as compared to vehicle-treated INS-1E cells (Figure 5B). We also evaluated the β-oxidation axis of the LDL particles through the expression of the Acetyl CoA carboxylase (ACC1) in INS-1E cells exposed to 2 mM nLDL or oxLDL for 48 h. As compared to control, nLDL induced a two fold increase in ACC1 mRNA expression, and oxLDL induced a 1.5 fold increase, suggesting that oxLDL are less metabolized than nLDL particles (Figure 5B). Inhibition of the carnitine palmitoyl transferase (CPT1) using etomoxir inhibits the lipid β-oxidation. In control conditions, a 48 h treatment with etomoxir (50 µM) had no impact on INS-1E cells viability (Figure 6A). In contrast, in presence of 2 mM nLDL or oxLDL, etomoxir significantly increased INS-1E cell apoptosis, suggesting that lipid metabolism may protect INS-1E cells against LDL toxicity (Figure 6A). On the contrary, the antioxidant N-acetyl-cystein (NAC 1 mM) partially prevented oxLDL-induced apoptosis (Figure 6A) as previously reported [19]. We tested the effect of lipoproteins on the production of reactive oxygen and/or nitrogen species (ROS/RNS), as well as superoxide anions (O_2_^−^) in living cells (Figure 6B–C). We observed that oxLDL (2 mM for 48 h), but not nLDL, stimulated the production of ROS/RNS (Figure 6B) and superoxide (Figure 6C). As a positive control, INS-1E cells were treated for 30 min with the ROS inducer Pyocyanin (200 µM). The antioxidant NAC (1 mM) efficiently prevented oxLDL-induced production of ROS/RNS. HDL, which has been shown to prevent oxidative stress in a variety of models, also significantly reduced oxLDL-induced ROS/RNS production (Figure 6B). Neither NAC nor HDL blocked oxLDL-induced generation of superoxide anions (Figure 6C). Etomoxir had no effect on basal or ox-LDL-induced ROS/RNS and superoxide production (Figure 6B–C). A 48 h treatment with oxLDL, but not nLDL, also stimulated the expression of the cytosolic Cu/Zn-superoxide dismutase (SOD1) (Figure 6D), but not the mitochondrial Mn-superoxide dismutase (SOD2) (data not shown). Etomoxir alone had no effect on SOD1 expression but stimulated SOD1 expression in presence of nLDL. However it did not exacerbate oxLDL-induced SOD1 overexpression (Figure 6D). In parallel, etomoxir treatment led to a significant increase in ICER-1 mRNA levels and enhanced the oxLDL-induced ICER-1 overexpression (Figure 6E). The effect of etomoxir on ICER-1 expression was associated with a decrease in Cx36 mRNA levels (Figure 6F). Since ceramide pathway has been proposed to play an important role in lipid toxicity, we tested the effect of the serine palmitoyltransferase inhibitor myriocin, which blocks ceramide synthesis, on cell viability and ICER-1 and Cx36 mRNA levels in INS-1E cells exposed to nLDL or oxLDL particles. Myriocin treatment (100 nM) had no effect on basal or nLDL-treated cell apoptosis (Figure 6G). However it significantly reduced oxLDL-induced apoptosis (Figure 6G). There was also a tendency to reduce oxLDL-induced ICER-1 overexpression (Figure 6H), which correlated with partly restored levels of Cx36 mRNA upon myriocin addition (Figure 6I).

**Figure 7:**
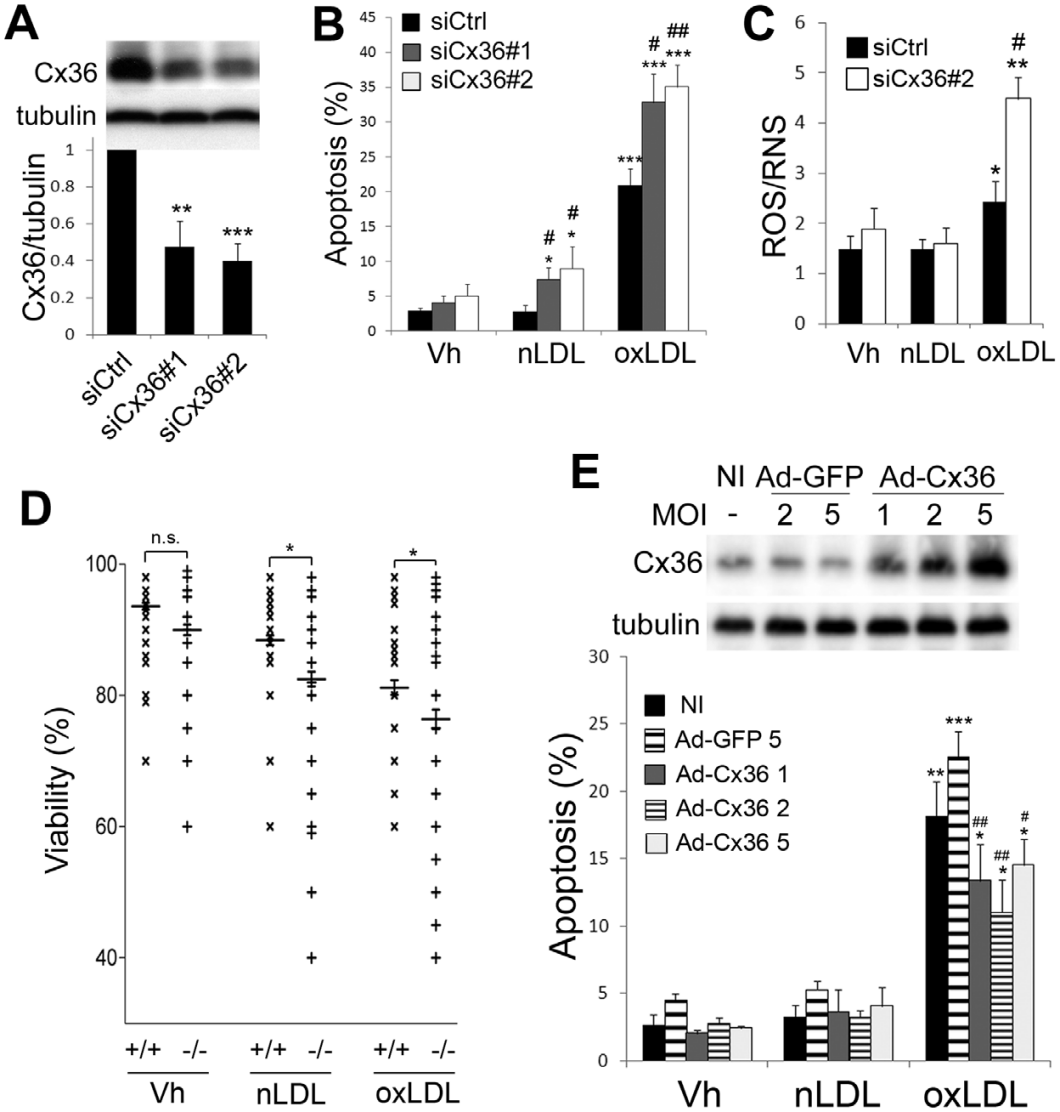
Cx36 overexpression protects INS-1E cells against oxLDL-induced apoptosis. (A–C) INS-1E cells were transfected with a control siRNA (siCtrl) or two different Cx36 siRNA (siCx36#1 and #2). (A) *Upper panel*: representative Western blot of Cx36 over tubulin expression. *Lower panel:* data are means ± SEM of 4 independent experiments. (B) Prevalence of apoptosis was evaluated by HO-PI staining after 48 h of exposure to 2 mM nLDL or oxLDL particles. (C) ROS/RNS production was evaluated in live cells after 48 h of exposure to 2 mM nLDL or oxLDL particles. (B–C) Data are means ± SEM of five experiments. *P<0.05, **P<0.01, ***P<0.001 vs. Vh condition.^ #^P<0.05 ^##^P<0.01 vs. siCtrl-transfected condition. (D) Islets from WT (+/+) or Cx36 KO (−/−) mice were exposed for 72 h to 2 mM nLDL or oxLDL particles. Islets viability was evaluated by HO-PI staining. Data are aligned dot plots of individual islet viability values from five animals per group (+/+ or −/−) and two separated LDL preparations. Horizontal bars show mean value ± SEM. n.s. (non-significant), *P<0.05 vs respective WT values (χ2 tests; see table S1). (E) INS-1E cells were infected or not (NI) with a control adenovirus (Ad-GFP) or a rat Cx36 adenovirus (Ad-Cx36) at various multiplicity of infection (MOI) as indicated. Cells were then exposed or not (Vh for Vehicule) for 48 h to 2 mM native (nLDL) or oxidized LDL (oxLDL). *Upper panel:* Representative WB of Cx36 over tubulin expression after infection. *Lower panel*: prevalence of apoptosis was evaluated by HO-PI staining. Data are mean ± SEM of at least 4 independent experiments. *P<0.05, **P<0.01, ***P<0.001 vs. respective Vh and nLDL conditions. ^#^P<0.05, ^##^P<0.01 vs. respective non-infected and Ad-GFP-infected conditions.

To elucidate the importance of Cx36 down-regulation in oxLDL-induced apoptosis, INS-1E cells were transiently transfected with two siRNAs directed against rat Cx36 (siCx36 #1 and #2). Both siRNAs decreased by about 60% Cx36 mRNA (data not shown) and protein levels (Figure 7A). We next evaluated the effect of the Cx36 knockdown on β-cell survival. As compared to cells transfected with a control siRNA (siCtrl), transfection with Cx36 siRNA#1 or #2 had no effect on basal apoptosis but rendered nLDL slightly toxic and aggravated apoptosis induced by a 48 h treatment with oxLDL (Figure 7B). Cx36 knock-down also aggravated oxLDL-induced ROS/RNS production (Figure 7C) but had no impact on basal or ox-LDL-induced superoxide production. To assess the relevance of theses observation in primary cells, pancreatic islets were extracted from WT (+/+) or Cx36 knock out (−/−) mice and treated for 72 h with 2 mM nLDL or oxLDL. Staining of islets with Hoechst-propidium iodine revealed that nLDL and, to a greater extend oxLDL particles, significantly reduced islets viability in WT and Cx36−/− islets (Figure 7D). As compared to WT(+/+) islets, Cx36−/− islets were sensitized to nLDL or oxLDL toxicity (see Table S1 for individual values and statistical analyses). To test whether Cx36 protects β-cell against oxLDL, INS-1E cells were infected with an adenovirus overexpressing rat Cx36 (Ad-Cx36) [5]. As compared to non-infected (NI) cells or cells infected with the control virus encoding GFP (Ad-GFP), Ad-Cx36-infected INS-1E cells displayed dose-dependently increased levels of Cx36 (Figure 7E upper panel). We next evaluated the effect of Cx36 overexpression on β-cell survival. As compared to non-infected (NI) or Ad-GFP-infected cells, Ad-Cx36-infected INS-1E cells displayed a 30% reduction in apoptosis following exposure to oxLDL particles (Figure 7E lower panel).

## Discussion

Obesity and more particularly, high levels of low density lipoprotein (LDL) particles, together with low levels of HDL particles, are important risk factors leading to the development of T2D and the metabolic syndrome [9], [10], [11], [12], [13]. The purpose of this study was to evaluate the impact of prolonged exposure to high levels of LDL-cholesterol on Cx36 expression since low Cx36 levels have been associated with reduced β-cell function and survival [3], [7]. The characterization of the ApoE−/− mice, which display drastically increased levels of circulating cholesterol [22], [23], [29], [39], revealed that high plasma LDL concentrations are associated with increased levels of ICER-1, and concomitant decreased levels of Cx36 in pancreatic islets. Using purified human LDL particles, we found that oxidized LDL (oxLDL) particles specifically drive the overexpression of the repressor ICER-1/ICER-1γ, which in turn binds to the Cx36 promoter, leading to down-regulation of Cx36 transcripts and protein expression levels. We also show that oxLDL particles are more toxic than native LDL (nLDL) particles because defective storage and metabolism. We further demonstrate that Cx36 overexpression partially protects β-cells against oxLDL-induced apoptosis.

We previously showed that mice fed a high fat diet express increased levels of ICER-1 and reduced levels of Cx36 [4]. These mice had elevated levels of circulating FFA, but they were also slightly hyperglycemic and hypercholesterolemic (total cholesterol) [4]. Here, we evaluated specifically the effect of cholesterol on Cx36 expression. The four months-old ApoE−/− mice used for this study had typically increased levels of circulating total cholesterol and no change in glycemia [40]. These mice also displayed slightly increased circulating levels of FFA (2 fold), and we cannot exclude that these FFAs contribute to Cx36 down-regulation in ApoE−/− mice as previously shown upon a prolonged high fat diet [4]. Despite the dramatic hypercholesterolemia, ICER-1 and Cx36 expression were not considerably changed in those mice. This is in accordance with our *in vitro* data showing that normal LDL particles are not deleterious to Cx36 expression in β-cells. Of note, Cx36 protein levels as assessed *in situ* by immunostaining were far more decreased in ApoE−/− animals compared to WT than at the mRNA level. This suggests that either Cx36 protein is further destabilized in ApoE−/− or reveals a biased assessment of Cx36 mRNA expression in freshly isolated islets due to the many artifacts (exposure to RNase, reducing agents, non linear RNA loss during islet and mRNA isolation…) and sampling bias (less than 1/3 of the existing islets can be isolated in mice as per the most efficient isolation procedures) which hinder analysis of gene expression from isolated islets.

The ApoE−/− mice have raised concerns owning to the very high “non-physiological” plasma cholesterol levels and the “quality” of lipoproteins [41], [42], as cholesterol is carried mostly in large VLDL and chylomicron remnants [40], whereas pro-atherogenic particles are thought to be mostly small-dense oxidized/glycated LDL particles [9], [43]. Yet, the ApoE−/− mice have been extensively used to study atherosclerotic lesions because they rapidly develop large plaque throughout the length of the arterial tree [40], [41], [42], suggesting that a significant fraction of the circulating lipoprotein have atherogenic properties. In addition, and in contrast with the ApoE−/− mice originally described [44], our ApoE−/− mice also had increased circulating levels of HDL particles, which have been shown to protect against the deleterious effects of oxLDL particles [18], [45], [46], [47], [48]. Altogether those limitations may account for the seemingly little impact of hypercholesterolemia on ICER-1 and Cx36 expression *in vivo.*

This study demonstrates that normal human LDL particles have no deleterious effects on β-cell survival whereas oxidized LDL particles have pro-apoptotic effects at fairly physiological concentration (2 mM), supporting the hypothesis that oxLDL particles greatly contribute to β-cell dysfunction and death in the pathophysiology of T2D. This is particularly important as small dense modified LDL particles are early markers of T2D [11], [14], [15]. The mechanisms responsible for the detrimental impact of oxLDL particles on β-cell function and survival are still poorly understood. The fact that saturated FFA and oxLDL both induce ICER-1 overexpression [4], [19] and Cx36 down-regulation ([4] and this study) strongly suggest that a similar mechanism is responsible for the effects of the lipids in both forms. The deleterious impact of saturated FFAs such as palmitate is due to accumulation of fatty AcylCoA entering non-oxidative toxic pathways of fatty acids metabolism, such as de novo ceramide formation which trigger oxidative stress in the mitochondria [38]. Our data indicate that nLDL particles stimulate lipid β-oxidation as assessed through Acc1 overexpression. On the other hand, oxLDL particles had only a marginal impact on ACC1 expression. Blocking lipid metabolism using the CPT1 inhibitor etomoxir [49] rendered native LDL particles toxic and aggravated the pro-apoptotic impact of oxLDL. Conversely, the serine palmitoyltransferase inhibitor myriocin, which blocks the synthesis of ceramide, partially prevented oxLDL-induced apoptosis, ICER-1 overexpression and Cx36 downregulation, suggesting that ceramide production is instrumental in oxLDL-induced apoptosis. Altogether those data suggest that the toxic effect of oxLDL may partly be due to a defect in metabolism, leading to oxidative stress, ICER-1 overexpression, Cx36 down-regulation and apoptosis. Oil red O staining data also revealed that both nLDL and oxLDL particles lead to the formation of lipid droplets in β-cells. However, nLDL formed abundant big regular round shaped lipid droplets whereas oxLDL formed fewer and smaller lipid droplets, suggesting that oxLDL are not as well stored in the form of lipid droplets as nLDL in β-cells. These observations are confirmed at the molecular levels by lower levels of the known marker of lipid droplets Plin1 [50] in cell exposed to oxLDL as compared to nLDL particles. Altogether these data suggest that nLDL are not toxic at this concentration (2 mM) because they are partly β-oxidized and partly stored in the form of neutral harmless lipid droplets. On the other hand, oxLDL particles are toxic due to both defective storage and β-oxidation, which may both result in accumulation of toxic lipid metabolites such as ceramides generating oxidative stress both in the form of ROS/RNS and superoxide anions, similarly to what has been shown with palmitate [49], [51].

Our oxidation protocol leads to the formation of mildly oxidized LDL [18], [19]. Therefore the oxLDL preparation probably still contains partially oxidized or native nLDL particles. Whether oxLDL particles themselves or nLDL particles remaining in the oxLDL preparation are responsible for the intermediate effect of oxLDL on β-oxidation or lipid droplets formation remains to be determined.

Our data indicate that oxLDL stimulate the production of ROS/RNS and superoxide species. ROS/RNS production can be averted by the antioxidant N-Acetyl cysteine (NAC), whereas superoxide production is not prevented. NAC partially protected cells against oxLDL toxicity, indicating that the ROS/RNS species are involved in oxLDL-induced apoptosis. We previously demonstrated that oxLDL-induced oxidative stress leads to ICER-1 overexpression [19]. Furthermore, blocking ICER-1 overexpression using a siRNA strategy protects β-cell against oxLDL-induced apoptosis [19]. However the exact mechanisms underlying the pro-apoptotic impact of ICER-1 is poorly understood. We recently showed that Cx36 protects mice against β-cell apoptosis induced by streptozotocin or alloxan, two models of induced Type1 Diabetes (T1D) [7]. Here, we observed that Cx36 knock-down or knock-out sensitized β-cells to oxLDL particles and that Cx36 overexpression partially protected β-cells from oxLDL-induced apoptosis. We further show that Cx36 knock-down aggravated the production of ROS/RNS, but not superoxide species, suggesting that Cx36 is able to alleviate (“dilute”) the oxidative stress upon oxLDL exposure. Thus intercellular communication may provide protection against pro-apoptotic stresses involved in the pathophysiology of T2D. Together with our previous studies showing that prolonged exposure to glucose and/or saturated FFA lead to ICER-1 overexpression and Cx36 down-regulation in β-cells [4], [8], this study underscores the importance of this particular mechanism in the pathophysiology of T2D. We further demonstrated that HDL counteracts the effect of oxLDL on Cx36 expression. This is in accordance with our previous finding that HDL particles prevent oxLDL-induced ICER-1 overexpression [19] and other studies showing that HDL particles are potent antioxidants with strong anti-apoptotic properties in a variety of models [18], [45], [46], [47], [48].

Further studies are required to identify other ICER-1 target genes with anti-apoptotic properties in β-cells. Given the prominent role of Cx36 mediated intercellular communication in β-cell function [6] and survival [7], we suggest that oxLDL-induced Cx36 down-regulation contributes to oxidative stress induced upon prolonged exposure to oxLDL, which is involved in β-cells dysfunction and apoptosis.

## Supporting Information

Figure S1 **Cx36 immunolabeling is decreased in the pancreatic islets of APOE−/− mice.** A) Cx36 immunolabeling and DAPI staining of WT and APOE−/− pancreas sections. *Upper panels*: black and white Cx36 signal provided by specific antibodies is seen as white spots all along the membrane of most islet cells. *Lower panels*: merged Cx36 (green) and DAPI (blue) staining. I: islet; E:exocrine tissue. The islet border is outlined by a dotted white line. Bars: 40 µm. B) Quantitative assessment of Cx36 immunostaining in WT and ApoE−/− mice. Data are mean ± SEM of 20 to 30 images from 2 distinct experiments and three animals in each group. *** p<0.001 in ApoE−/− vs WT mice.
(TIF)

Table S1 **Statistic table showing the ranking of the viability values obtained for the experiments showed in** **Figure 7D** **and used to perform statistical analysis.**
(DOCX)

Methods S1 **Cx36 immunostaining, image processing and quantification.**
(DOCX)
