# Supplementary figures and images for: Reduction of Connexin36 Content by ICER-1 Contributes to Insulin-Secreting Cells Apoptosis Induced by Oxidized LDL Particles
Source: PLoS One. 2013 Jan 30;8(1):e55198. doi: 10.1371/journal.pone.0055198 (PMC3559396; doi:10.1371/journal.pone.0055198)

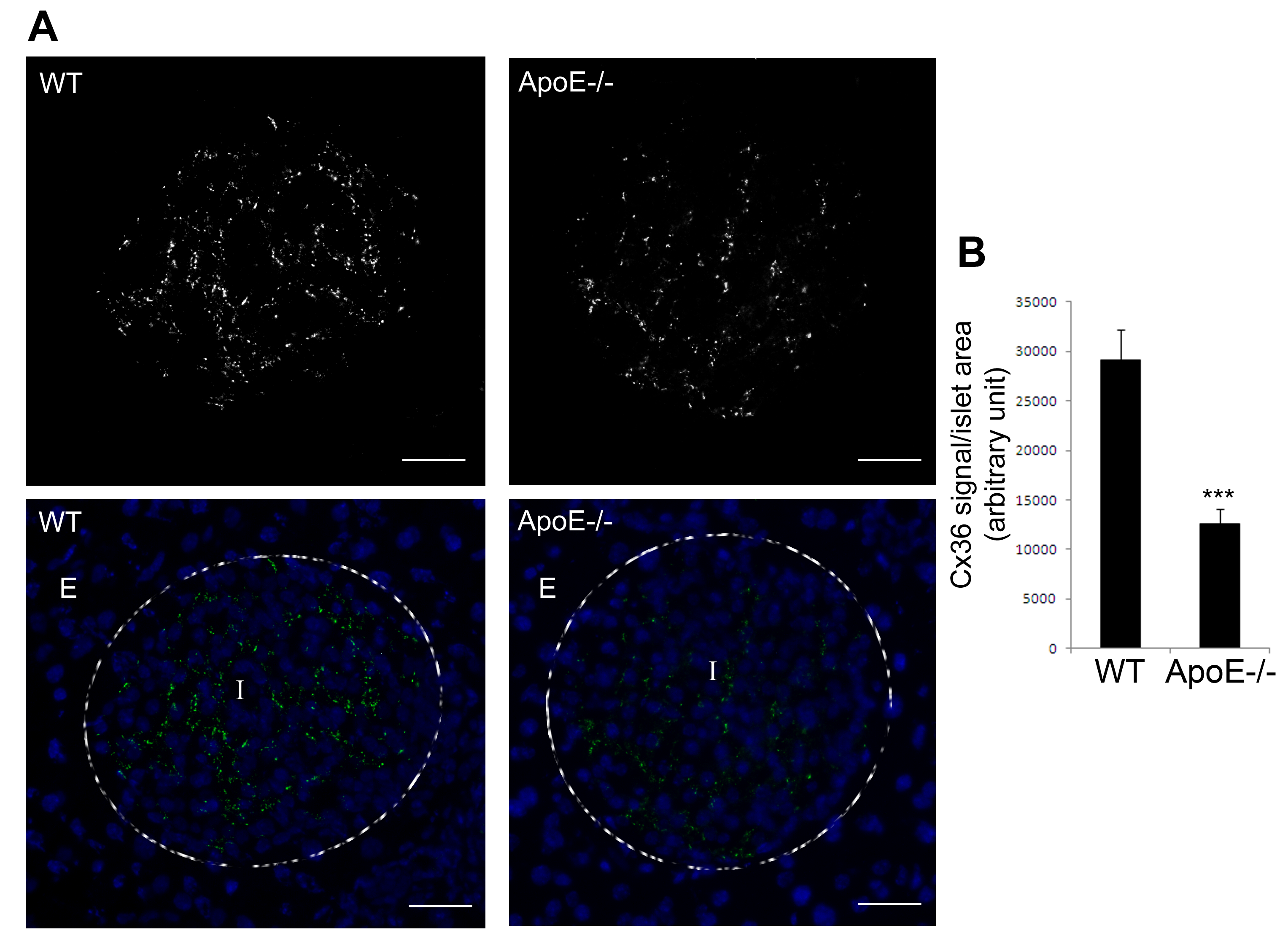

Supplement: Figure S1 — Cx36 immunolabeling is decreased in the pancreatic islets of APOE−/− mice. A) Cx36 immunolabeling and DAPI staining of WT and APOE−/− pancreas sections. Upper panels: black and white Cx36 signal provided by specific antibodies is seen as white spots all along the membrane of most islet cells. Lower panels: merged Cx36 (green) and DAPI (blue) staining. I: islet; E:exocrine tissue. The islet border is outlined by a dotted white line. Bars: 40 µm. B) Quantitative assessment of Cx36 immunostaining in WT and ApoE−/− mice. Data are mean ± SEM of 20 to 30 images from 2 distinct experiments and three animals in each group. *** p<0.001 in ApoE−/− vs WT mice. (TIF) [file pone.0055198.s001.tif]
