# Supplementary material for: Reduction of Connexin36 Content by ICER-1 Contributes to Insulin-Secreting Cells Apoptosis Induced by Oxidized LDL Particles
Source: PLoS One. 2013 Jan 30;8(1):e55198. doi: 10.1371/journal.pone.0055198 (PMC3559396; doi:10.1371/journal.pone.0055198)
Supplement: Table S1 — Statistic table showing the ranking of the viability values obtained for the experiments showed in Figure 7D and used to perform statistical analysis. (DOCX) [file pone.0055198.s002.docx]

| **islets** | **viability (%)** | **30-40** | **41-50** | **51-60** | **61-70** | **71-80** | **81-90** | **91-100** | **χ^2^** |
| --- | --- | --- | --- | --- | --- | --- | --- | --- | --- |
| **WT (+/+)** | **Vh** |  |  | 0 | 1 | 5 | 16 | 50 |  |
|  | **natLDL** |  |  | 1 | 5 | 7 | 45 | 27 | ******* |
|  | **oxLDL** |  |  | 13 | 20 | 25 | 31 | 24 | *****^, #^** |
|  |  |  |  |  |  |  |  |  |  |
| **Cx36KO**  **(-/-)** | **Vh** | 0 | 0 | 0 | 2 | 8 | 37 | 51 |  |
|  | **nat LDL** | 1 | 1 | 7 | 11 | 21 | 33 | 26 | ******* |
|  | **oxLDL** | 2 | 7 | 13 | 23 | 20 | 20 | 18 | *****, ^#^** |
|  |  |  |  |  |  |  |  |  |  |
| **Vh** | **+/+** |  |  |  | 1 | 5 | 16 | 50 |  |
|  | **-/-** |  |  |  | 2 | 8 | 37 | 51 | **n.s** |
| **natLDL** | **+/+** | 0 | 0 | 1 | 5 | 7 | 45 | 27 |  |
|  | **-/-** | 1 | 1 | 7 | 11 | 21 | 33 | 26 | **^$^** |
| **oxLDL** | **+/+** | 0 | 0 | 13 | 20 | 25 | 31 | 24 |  |
|  | **-/-** | 2 | 7 | 13 | 23 | 20 | 20 | 18 | **^$^** |

**Supplemental Table S1:** Cell viability was measured in intact islets using HO-PI staining in the absence (Vh) or after 72-h exposure to natLDL or oxLDL particles. Data are individual islet viability values from five animals per group, 20 islets per conditions (WT (+/+) or Cx36KO (-/-)) and two separated LDL preparations. Viability values were ranked and evaluated by a non-parametric χ^2^ test. *** P<0.001 vs. Vh condition; # P<0.01 vs. natLDL condition; $ p<0.05 in Cx36-/- compared to WT (+/+) mice.
