# Supplementary material for: Reduction of Connexin36 Content by ICER-1 Contributes to Insulin-Secreting Cells Apoptosis Induced by Oxidized LDL Particles
Source: PLoS One. 2013 Jan 30;8(1):e55198. doi: 10.1371/journal.pone.0055198 (PMC3559396; doi:10.1371/journal.pone.0055198)
Supplement: Methods S1 — Cx36 immunostaining, image processing and quantification. (DOCX) [file pone.0055198.s003.docx]

**Supporting Methods S1**

**Cx36 immunostaining, image processing and quantification**

Cx36 immunofluorescent staining was performed as previously described [[1](#_ENREF_1),[2](#_ENREF_2)] on frozen sections of pancreas from 3 ApoE-/- and 3 cognate controls, using a polyclonal rabbit anti-Connexin 36 antibody (Life Technology Europe, Zug, Switzerland). Quantitative assessment of the membrane spots immunostained by the Cx36 anitbodies was performed using the ImageJ software. Brielfly, 20-30 images from at least 6 islets per animal, were first converted to a 32 bit format and subjected to background substraction. Signal to noise ratio was determined by applying the MaxEntropy thresholding method. A binary image was then created and a region of interest (ROI) corresponding to a pancreatic islet was created. The average pixel intensity of signal in each ROI was normalized to the area of each ROI (islet area). Values were obtained from two distinct experiments and three animals for each group.

1. Allagnat F, Martin D, Condorelli DF, Waeber G, Haefliger JA (2005) Glucose represses connexin36 in insulin-secreting cells. J Cell Sci 118: 5335-5344.

2. Klee P, Allagnat F, Pontes H, Cederroth M, Charollais A, et al. (2011) Connexins protect mouse pancreatic beta cells against apoptosis. J Clin Invest 121: 4870-4879.
